# The Potential Allergic Role of Mast Cells to Orthodontic Appliance in Orthodontic-Induced Gingival Enlargement

**DOI:** 10.3290/j.ohpd.c_2801

**Published:** 2026-09-04

**Authors:** Amal Ali Ibrahim, Omneya Emam Ahmed, Gihan A. Balbola, Amany M. Taha, Doaa Adel Habba, Basma Badr Hasaneen, Naglaa S. El-Kilani

**Affiliations:** a Amal Ali Ibrahim Associate Professor of Oral Medicine, Periodontology, Oral Diagnosis and Dental Radiology, Faculty of Dental Medicine for Girls, Al-Azhar University, Cairo, Egypt. Conducted the clinical part of the experiment, participated in data and statistical analysis, prepared the figures, interpreted the results and drafted the main manuscript.; b Omneya Emam Ahmed Associate Professor of Oral Medicine, Periodontology, Oral Diagnosis and Radiology Department, Faculty of Dental Medicine for Girls, Al-Azhar University, Cairo, Egypt. Conducted the clinical part of the experiment, participated in data and statistical analysis, prepared the figures, interpreted the results and drafted the main manuscript; revised and edited the final version of the manuscript.; c Gihan A. Balbola Associate Professor of Oral and Dental Pathology, Faculty of Dental Medicine for Girls, Al-Azhar University, Cairo, Egypt. Conducted the histological part of the experiment, participated in data and statistical analysis, prepared the figures, interpreted the results and drafted the main manuscript.; d Amany M. Taha Associate Professor of Oral and Dental Pathology. Faculty of Dental Medicine for Girls, Al-Azhar University, Cairo, Egypt. Conducted the histological part of the experiment, participated in data and statistical analysis; e Doaa Adel Habba Assistant Professor of Oral Pathology, Department of Oral and Maxillofacial Surgery and Diagnostic Sciences, Faculty of Dentistry, Najran University, Najran, Kingdom of Saudi Arabia. Lecturer of Oral and Dental Pathology. Faculty of Dental Medicine for Girls, Al-Azhar University, Cairo, Egypt. Conducted the histological part of the experiment, prepared the figures, interpreted the results and drafted the main manuscript; revised and edited the final version of the manuscript.; f Basma Badr Hasaneen Lecturer of Orthodontics, Faculty of Dental Medicine for Girls, Al-Azhar University, Cairo, Egypt. Conducted the clinical part of the experiment, participated in data and statistical analysis.; g Naglaa S. El-Kilani Professor of Oral Medicine, Periodontology, Diagnosis, and Radiology Department, Faculty of Dental Medicine for Girls, Al-Azhar University, Egypt. Conceived and designed the idea of the experiment, revised and edited the final version of the manuscript.

**Keywords:** allergy, c-kit, gingival enlargement, hypersensitivity reaction, mast cells, orthodontic appliance, toluidine blue.

## Abstract

**Background:**

Gingival mast cells (MCs) are strategically scattered throughout connective tissue and adhere to the endothelium to convert local impulses into systemic signals and attract additional immune effector cells. Numerous mediators, such as histamine, cytokines, and proteases, modulate these interaction pathways.

**Objectives:**

The purpose of this study was to determine and compare MC count in the gingival enlargement tissue of patients receiving orthodontic treatment versus patients having plaque-induced gingival enlargement without orthodontic treatment, utilising hematoxylin and eosin (H&E) staining, toluidine blue (TB), and CD117 (c-kit) immunohistochemistry (IHC) as a specific marker for MC identification.

**Methods and Materials:**

This study involved 30 patients who were divided into three groups: Group 1 included 10 patients with clinically healthy gingival tissue that intentionally needed crown lengthening. Group 2 included 10 cases of plaque-induced gingival enlargement in individuals without orthodontic treatment. Group 3 included 10 cases of orthodontic gingival enlargement in individuals receiving orthodontic treatment. Patients received non-surgical periodontal therapy and oral hygiene instructions. After the plaque control phase, with a Gingival Index (GI) = 0 and no Bleeding on Probing (BoP), the gingiva was surgically treated. During the surgical procedure, the excised gingival margin was collected for histological examination with H&E, TB special stain, and c-kit to count the MCs.

**Results:**

The gingiva of patients in group 3 was characterised by more frequent epithelial changes (mild hyperplasia of the basal layer and spongiosis), a stronger degree of inflammation, including band-like inflammatory infiltrates and individual inflammatory cells, and a milder degree of fibrosis, compared to group 2. In addition, a significant increase in the number of MCs was observed in group 3 compared with groups 2 and 1. c-kit was a more specific marker for MCs than for TB.

**Conclusion:**

Orthodontic-induced gingival enlargement showed a significantly higher number of mast cells than plaque-induced gingival enlargement, and this involvement of MCs, particularly, may be associated with mast cell activation and degranulation. These findings may suggest an immunological role for MCs in the pathogenesis of orthodontically induced gingival enlargement.

During orthodontic treatment, patients often experience gingival enlargement, which may be caused by poor biofilm control, certain medications, or the irritating effects of nickel or metal allergy.^39^ Nickel and titanium are the constituent elements of orthodontic alloys.^9^ It has been noted that wearing certain metal appliances may alter electrolyte levels in saliva, raising salivary nickel or chromium levels.^12^


Due to the oral corrosion of alloys during orthodontic treatment, allergic reactions from titanium and/or nickel can occur.^9^ It is worth noting that nickel ions are among the most critical metals implicated in contact dermatitis and, regrettably, can cause oral contact allergies.^22^


 Mast cells (MCs) are tissue-specific cells, descended from the myeloid lineage. Progenitor cells differentiate into mature mast cells under the influence of stem cell factors secreted by several cell types.^5^ MCs can influence innate and adaptive immune responses due to their anatomical distribution and structural linkages. In chronic inflammation, MCs produce a range of bioactive amines, chemokines, cytokines, proteoglycans, and growth hormones that modulate diverse functions.^35^


In addition, MCs are important in allergic responses.^4^ However, to facilitate cell degranulation and the excretion of their mediators, mast cell activation is required.^35^


MCs stain with basic dyes such as toluidine blue (TB) and methylene blue. MCs are identified by the presence of MC-specific enzymes, mainly tryptase and chymase, which are assessed by immunohistochemistry.^11^ MCs express a wide variety of receptors, including stem cell factor receptor and CD117 (c- kit), which persists in mature MCs.^28^


There is little scientific evidence explaining why patients undergoing orthodontic treatment are more prone to gingival overgrowth than healthy patients. Also, the specific mechanism by which gingival enlargement develops is still unclear. Thus, the purpose of this study was to determine and compare MC counts in the gingival enlargement tissue of patients receiving orthodontic treatment versus patients having plaque-induced gingival enlargement without orthodontic treatment, utilising hematoxylin and eosin (H&E) staining, toluidine blue (TB), and c-kit immunohistochemistry (IHC) as a specific marker for MC identification.

## Methods and Materials

### Study Population

The research was approved by the Research Ethics Committee, Faculty of Dental Medicine for Girls, Al-Azhar University (Code: REC-PD-25-04). Patients were chosen from the outpatient clinics of the oral medicine and periodontology department at the Faculty of Oral and Dental Medicine, Al-Azhar University, Cairo, Egypt, for this single-blind clinical study. The purpose of the study was explained to each patient, and informed written consent was obtained from the included patients before the treatment. The sample size was determined using G*Power version 3.1.9.7 (University of Düsseldorf, Düsseldorf, Germany) with α = 0.05 and a power of significance of 95%, and a total of thirty patients were needed, with ten patients in each group, based on the results of the previous research.^25^


### Grouping of Patients

Thirty patients, ages 22 to 35, participated in the current study. They were categorised into three groups. Group 1 included 10 patients with normal gingival tissue that intentionally needed crown lengthening. Group 2 included 10 cases of plaque-induced gingival enlargement in individuals without orthodontic treatment. Group 3 included 10 cases of orthodontic gingival enlargement in individuals receiving orthodontic treatment.

Based on the following inclusion criteria, a skilled periodontist validated the diagnosis of gingival enlargement: all patients showed keratinised, non-inflammatory fibrous gingival enlargement, no clinical attachment loss (CAL = 0), and, according to Loe and Silness (1963),^18^ the Gingival Index (GI) equalled 0 and no Bleeding on Probing (BoP). Furthermore, there was no radiological evidence for alveolar bone loss. The patients with idiopathic or drug-induced gingival enlargement, pregnant or lactating women, and active periodontal disease were excluded according to the study eligibility criteria.

The orthodontic patients underwent meticulous non-surgical periodontal therapy to maintain a plaque index score of 0 during the study period to prevent exaggerated gingival enlargement caused by dental plaque. Before the gingival tissues were obtained, all patients in all groups received non-surgical periodontal therapy, including oral hygiene instructions, scaling and plaque control measures for 2 weeks. For group 1, gingival tissue was taken from patients following tooth extraction for orthodontic purposes or crown lengthening. Gingivectomy was carried out in groups 2 and 3 for patients whose gingival enlargement remained following non-surgical periodontal therapy. During the surgical procedure, the excised gingival margin was collected for histological examination with H&E, TB stain, and c-kit to count MCs.

### Tissue Staining Techniques

After being promptly preserved in 10% neutral buffered formalin, the gingival tissues were divided into sections that were 5 µm thick to undergo three staining methods. To confirm the diagnosis of gingival hyperplasia histologically, the sections were stained with H&E according to standard protocol. Pathohistological changes in the gingiva were analysed, including epithelial and stromal changes, intensity of fibrosis, and intensity and localisation of the inflammatory infiltrate. Following verification of gingival hyperplasia, TB staining and c-kit immunohistochemical staining were performed on the remaining two sections.

#### Staining with TB

After being deparaffinised, sections were dehydrated with water. TB staining was performed with 1% TB for 30 min, followed by dilution in phosphate-buffered saline (PBS) (pH 4–6) for 45 s. The sections were meticulously blotted and rapidly dehydrated using 70% and 96% ethanol, then 100% alcohol, and finally xylene, and then mounted in synthetic resin and observed under a binocular microscope. Using a light microscope, a pathologist who was blind to the clinicopathological information for each specimen examined every slide. Using a magnification of ×400, the number of MCs was assessed in ten microscopic regions in the hotspot and then calculated as the total number of MCs in 10 high-power fields, and the average number of MCs in each group was recorded.^11^


#### Immunohistochemical staining

On electrically positively charged glass slides, 5 μm sections were placed, deparaffinised with xylene, and then rehydrated in progressively decreasing ethanol concentrations before being washed with PBS. Soaking in 0.3% H_2_O_2_ to inhibit endogenous peroxidase. Slices were placed in a glass jar with 0.01M sodium citrate buffer (pH 6.0) for antigen retrieval.

Polyclonal rabbit anti-human c-kit staining (CD117 Diagnostic Biosystems, 6616 Owens Drive, Pleasanton, CA, 94588) and the universal kit were used for detection. After antigen-antibody visualisation using diaminobenzidine in PBS containing 40% H_2_O_2_, sections were counterstained with Mayer’s hematoxylin and mounted. On completion of IHC staining, a light microscope with ×400 was used to count the number of MCs in 10 high‑power fields in the highest-density field (hot spot). The number of MCs in each field was counted. Ten fields with the highest number of MCs were selected, and the average number of MCs in each group was recorded.^21^


### Statistical Analysis

Numerical data showed a parametric distribution, so they were represented by mean and standard deviation values. Two-way ANOVA and post hoc tests for the comparison between different groups.

## Results

A comparison of mast cell staining results using H&E, TB special stain, and c-kit immunostaining can provide insight into mast cell involvement in gingival health and pathology, especially during orthodontic treatment.

### Pathohistological Features of Gingival Tissue

The gingival tissues of group 1 (Fig 1a) exhibited almost normal histological architecture with minimal inflammatory infiltrates in connective tissue (CT), compared to groups 2 and 3. While in group 3, the gingiva tissue, showed more frequent epithelial changes (mild hyperplasia of the basal layer and spongiosis), a stronger degree of inflammation including (band-like inflammatory infiltrates and individual inflammatory cells) in CT and a milder degree of fibrosis (Fig 1b) compared to group 2 that showed less pronounced epithelial hyperplasia, multifocal inflammation in CT, moderate and severe fibrosis (Fig 1c).

**Fig 1a to c Fig1atoc:**
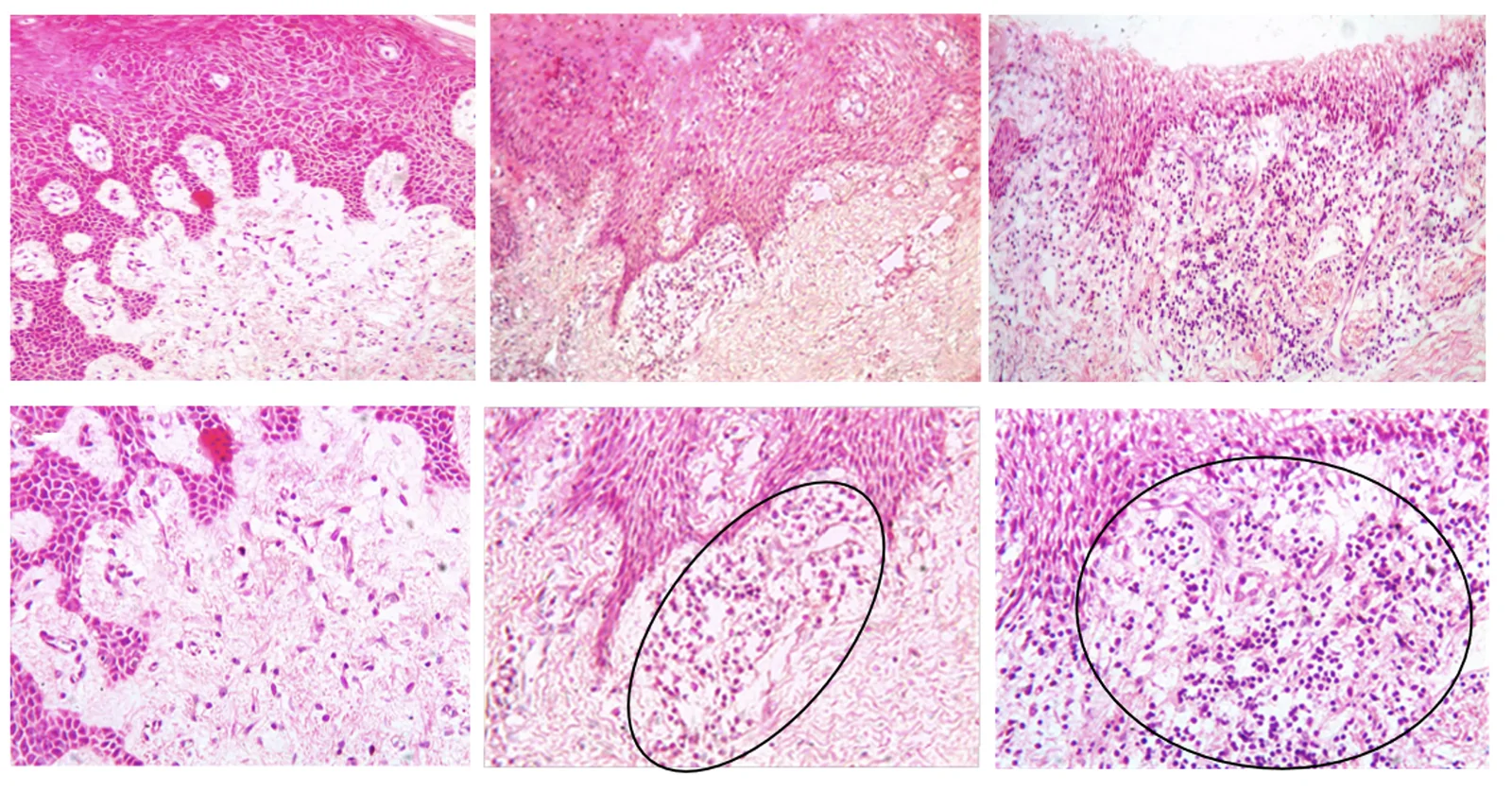
A photomicrograph of gingival tissue stained with Hematoxylin and Eosin in different study groups: (a) group 1, the CT revealed minimal spread of inflammatory cells between the collagen fibres (black circle). (b) The circled CT area of group 2 showed focal inflammation between dense collagen fibres. (c) Group 3 showed band-like inflammatory infiltrates and individual inflammatory cells in between discrete collagen fibres at a magnification of ×100 (A, B, and C) and ×200 (a, b, and c).

### Distribution of TB-Stained MCs

In group 1, the number of MCs was few, the density was relatively low, and the MCs were uniformly distributed within the CT (Fig 2A, a). Group 2 showed an increase in the number of and staining intensity of granulated MCs, which appear as discrete, rounded or oval, purple-blue cells (Fig 2b). Moreover, group 3 exhibited a much higher number of MCs than groups 1 and 2; additionally, an increase in the number of degranulated cells and the presence of reddish-purple granules scattered throughout the epithelium and CT (Fig 2c).

**Fig 2a to c Fig2atoc:**
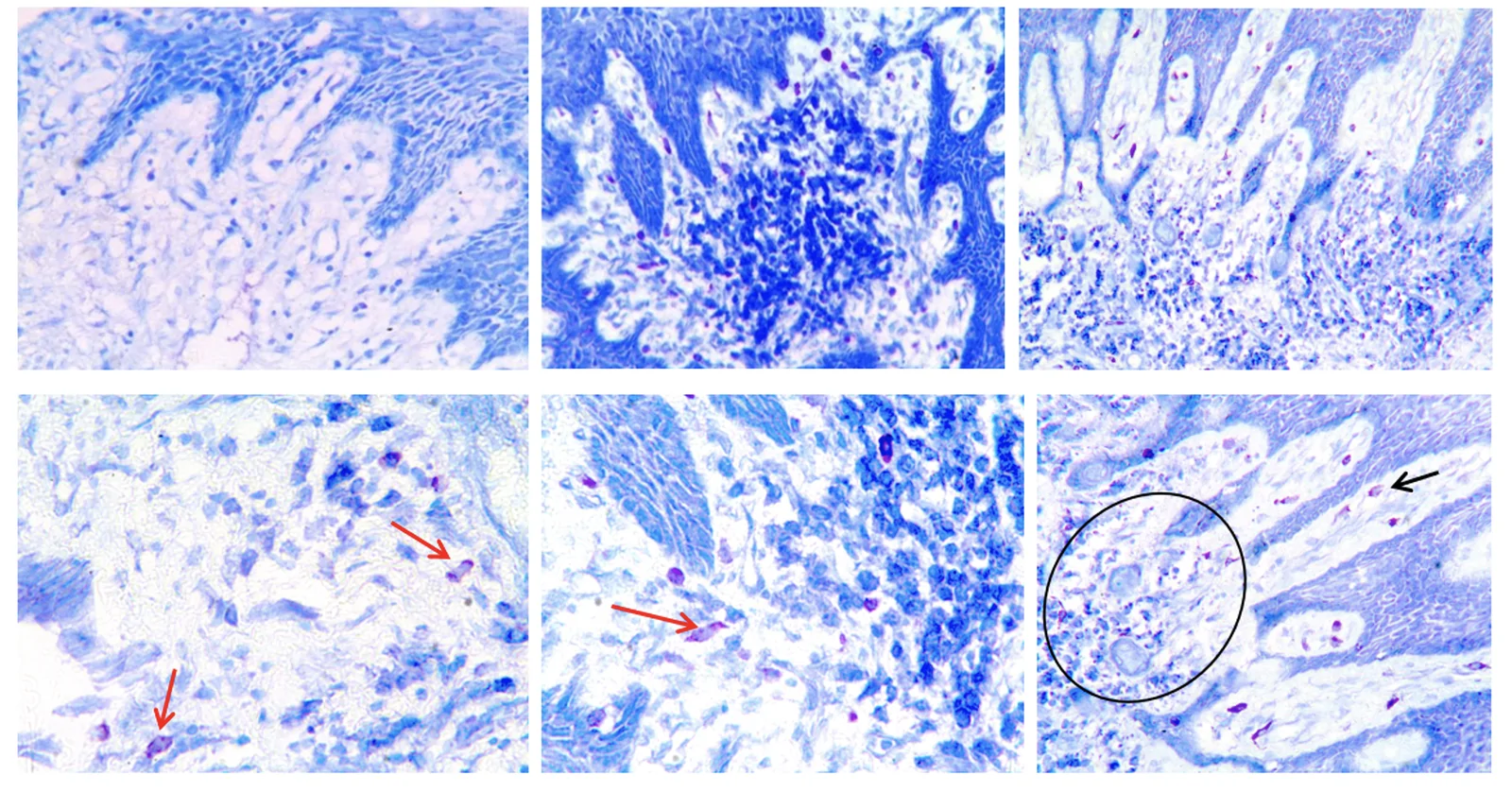
A photomicrograph of gingival tissue stained with toluidine blue showed mast cells in different study groups: (a) group 1 showed few granulated mast cells in normal gingiva (red arrow). (b) Group 2 showed many granulated mast cells that appear as oval or rounded purple-blue cells (red arrow), mainly in the CT. (c) Group 3 showed a large number of granulated (red arrow) and degranulated mast cells (black arrow) with diffuse reddish-purple metachromatic granules (black circle) juxtaepithelial and in the CT at a magnification of ×200 (A, B and C) and ×400 (a, b and c).

#### C-kit expression in mast cells

Immunostaining of group 1 revealed that MCs express low c-kit intensity compared to groups 2 and 3, which verifies their presence in healthy gingival tissue (Fig 3a). Group 2 showed an increase in c-kit expression in mast cells (Fig 3b), and group 3 showed marked c-kit expression, which suggests their potential response to the degranulation of the MCs (Fig 3c).

**Fig 3a to c Fig3atoc:**
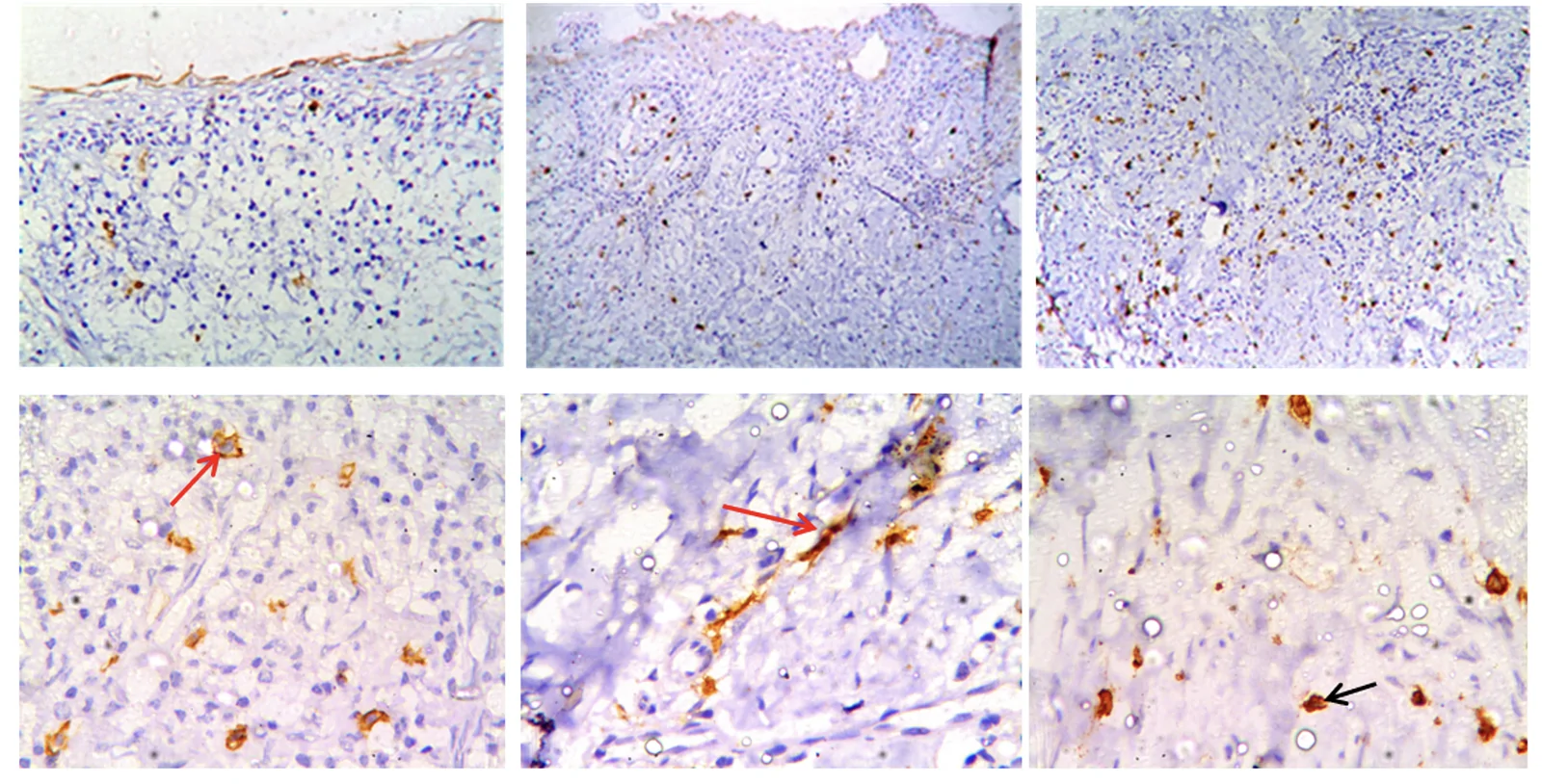
Immunohistochemically C-KIT-stained mast cells in different study groups: (a) group 1 showed few granulated mast cells that appear brown in normal gingiva (red arrow). (b) Group 2 showed a small, discrete distribution of mast cells mainly granulated in the C.T (red arrow). (c) Group 3 showed a large number of mast cells mainly degranulated (black arrow). With diffuse distribution near the epithelium and in the connective tissue at a magnification of ×200 (A, B, and C) and ×400 (a, b, and c).

#### Quantitative assessment of mast cells across three groups

The quantitative evaluation of MCs in the study groups, based on TB staining, showed that group 3 had the highest mean number of MCs (5.20 ± 1.23), followed by group 2 (3.80 ± 0.50) and group 1 (1.60 ± 0.44). When evaluating MCs stained with c-kit expression, there was a highly statistically significant highest mean value of the number of MCs stained in group 3, which was 15.20 ± 2.35, followed by group 2, which was 8.20 ± 1.55, and group 1, which was 4.20 ± 1.61. The number of MCs stained in c-kit showed a highly statistically significant mean value compared to TB in each group (Fig 4 and Table 1).

**Fig 4 Fig4:**
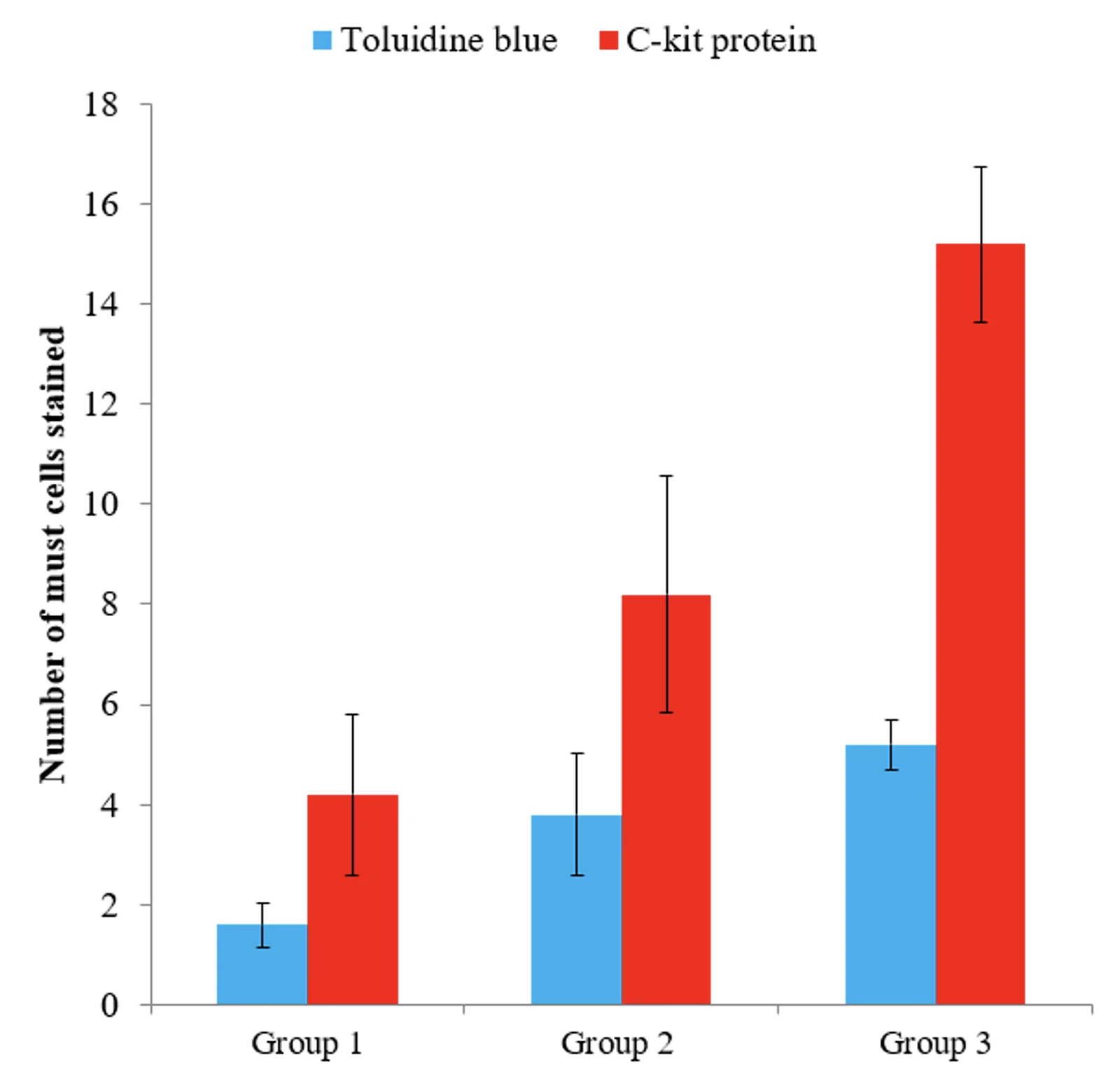
The mean ± SD values of the number of mast cells stained in three groups.

**Table 1 Table1:** The mean ± SD value of the number of stained MCs for the comparison between three groups

	TB Mean ± SD	C-kit protein Mean ± SD	*p* value
Group 1	1.60 ± 0.44^Bb^	4.20 ± 1.61^Ac^	**0.001***
Group 2	3.80 ± 0.50^Bc^	8.20 ± 1.55^Ab^	**0.001***
Group 3	5.20 ± 1.23^Ba^	15.20 ± 2.35^Aa^	**0.001***
*p* value	0.001*	0.001*	
Significance level *p* ≤ 0.05, *significant. Different capital letters indicate significant difference at (*p* < 0.05) among means in the same row. Different small letters indicate significant difference at (*p* < 0.05) among means in the same column.			

## Discussion

Orthodontic patients typically exhibit several oral clinical symptoms, including gingival enlargement, sensitivity, and an elevated risk of caries.^8^ For management to be effective, the cause of the enlargement must be accurately determined.^2^


MCs are present subepithelial and intraepithelial in the gingival CT, often in the vicinity of endothelial cells. MCs are shown to be more numerous in inflamed gingiva.^38^ MCs are essential in the pathophysiology of several diseases, including gingival irritational fibromatosis, chronic hypertrophic dermatitis, and oral squamous cell carcinoma.^17^ Since MCs and fibroblasts share staining features, it is challenging to visualise them with H and E stain, the standard stain used in labs to diagnose reactive lesions. For MC identification, toluidine blue has been widely used due to its ability to easily distinguish mast cells against a blue background, its metachromatic staining properties for heparin, and its low cost.^29^ However, immature or deregulated mast cells are not stained, making this approach less accurate.^3^ Therefore, we use c-kit immunohistochemistry in conjunction with TB to identify MCs with high specificity and accuracy.

Moreover, c-kit plays an important role in MC degranulation. The binding of stem cell factor (SCF) to the c-kit receptor enhances FcεRI aggregation at the mast cell surface, leading to MC degranulation. In other words, the c-kit ligand increased IgE-dependent degranulation.^33,37
^


Pathohistological features of group 2 showed less pronounced epithelial hyperplasia, multifocal inflammatory infiltrates with plasma cells and lymphocytes, and moderate and severe fibrosis. Histopathological analyses of the gingival enlargement in group 2 were also performed in another study, which showed that dental biofilm-induced gingival enlargement is characterised by dense, more regularly distributed subepithelial collagen that limits the zone of chronic inflammatory cells.^7^


In group 3, the gingiva showed more frequent epithelial changes (mild basal-layer hyperplasia and spongiosis), a stronger degree of inflammation, including band-like infiltrates and individual inflammatory cells, and a milder degree of fibrosis.

This histological finding is similar to a previous study that defined the appearance of the inflammatory infiltrate, and the presence of band-like inflammatory infiltrates in sensitised orthodontic gingival enlargement may reflect the organisation of inflammatory cells.^39^ Moreover, similar to those observed in the lichenoid reaction. Earlier studies have also reported band-like lichenoid infiltrate in oral contact allergies.^16^


Quantitative assessment of MCs revealed that group 2 in this study had a significantly higher count of MCs stained by TB and c-kit than group 1. MCs participate in inflammatory conditions by selectively releasing mediators without degranulation, unlike in allergic reactions.^32^ This particular mediator, including serotonin that is excreted from secretory granules, without histamine.^30,31
^


MCs activated by IL-1 in response to bacterial lipopolysaccharide released IL-6 without degranulation,^14^ and prostaglandin E2 induced the release of vascular endothelial growth factor 1 without degranulation.^23^ Additionally, stromal cell-derived factor-1α (SDF-1α) selectively induced IL-8 from MCs without degranulation.^19^


In similar findings, Huang et al (2013) and Marjanović et al (2016) discovered that MCs progressively increased from healthy gingiva to severe gingivitis.^10,20
^ Additionally supporting our findings, Kamal et al (2011) discovered that when stained with 1% TB, the number of MCs in granuloma gravidarum was higher than in normal oral mucosa.^13^ These findings are consistent with a prior study that found that, compared with normal gingiva, the mean number of MCs was higher in oral reactive lesions, with considerably higher levels of inflammatory fibrous hyperplasia and irritational fibroma.^8^


Compared with group 2, the number of MCs was significantly higher in group 3 (5.20 ± 1.23), followed by group 2 (3.80 ± 0.50), with a marked increase in the number of degranulated cells and reddish-purple granules. Regarding the immunohistological findings, the highest mean number of stained MCs was observed in group 3 (15.20 ± 2.35), followed by group 2 (8.20 ± 1.55) and group 1 (4.20 ± 1.61).

Interestingly, the number of MCs in specimens stained with TB was lower than that in c-kit immunostained specimens. The reason for the disparity in effectiveness between the two methods is that, while TB uses the principle of metachromatic staining, after granules are expelled from the cell, they lose their metachromatic properties and stain pink with toluidine blue, resulting in a lower MC count. C-kit immunohistochemical staining uses the principle of an antigen-antibody reaction and thus accurately detects both granulated and degranulated MCs.^15^


In this study, patients undergoing orthodontic treatment had the highest number of mast cells in the gingival tissue. This could be explained by nickel’s ability to irritate and possibly accumulate in the connective and epithelial tissues of the mouth mucosa during orthodontic therapy.^34^ Continuous low concentrations of nickel released throughout the presence of orthodontic wire in saliva trigger autocrine activation of keratinocytes, which encourages their proliferation.^6^


MC function during T-cell-mediated immune responses is contingent on the intensity of these responses. Additional factors that affect this intensity include the concentration of the allergen or hapten, the adjuvant or solvent used to sensitise and elicit the immune response, and the housing conditions, which affect the activation status of memory cells, particularly antigen-presenting cells.^36^ Thus, nickel, which primarily activates the Th1/Th17 and Th22 components, may have a substantial impact on the development of gingival enlargement in orthodontic patients^24^ through a delayed hypersensitivity reaction mediated by mast cells.

Furthermore, MC degranulation may also have contributed to the hyperactive MCs’ production of mediators like histamine, heparin, and TNF-α, which can affect fibroblast proliferation, ECM synthesis, and degradation; TNF-α also increases the expression of RANTES and C-C chemokine receptor Type 1, which further degranulates MCs,^26^ which can cause signs and symptoms similar to those of an allergic reaction.

Additionally, MCs’ effects in anaemia of chronic disease (ACD) have been studied using models of repeated antigen challenge. In this case, MCs alter the delayed response to an immediate response and accumulate in a manner dependent on L-selectin and ICAM-1.^27^


## Conclusion

Orthodontic-induced gingival enlargement showed a significantly higher number of mast cells than plaque-induced gingival enlargement, and this involvement of MCs, particularly, may be associated with mast cell activation and degranulation. These findings may suggest an immunological role for MCs in the pathogenesis of orthodontically induced gingival enlargement. Future studies investigating metal hypersensitivity and the use of alternative orthodontic materials are warranted to determine whether such approaches may help reduce the risk of gingival enlargement during orthodontic treatment.

### Limitation

Further studies are needed to perform functional assays of mast cells to determine mediator release. The involvement of T lymphocytes and their interaction with mast cells in the pathogenesis of orthodontic-induced gingival enlargement should also be assessed.

### Acknowledgements

The authors would like to express their gratitude to Professor Naglaa El-Kilani, Professor of Oral Medicine and Periodontology, Oral Diagnosis, and Dental Radiology at Al-Azhar University for Girls, Cairo, Egypt, for her efforts and support throughout the study.

This work was performed at the Faculty of Dental Medicine for Girls, Al-Azhar University, Cairo, Egypt.

#### Declaration

##### Ethics approval and consent to participate

The present study was conducted according to the guidelines of the Declaration of Helsinki and was approved by which was approved by the Research Ethics Committee of the Faculty of Dental Medicine for Girls at Al-Azhar University, Egypt (REC-PD-25-04).

##### Informed consent statement

Informed consent was obtained from all subjects involved in the study.

##### Data availability

The data supporting the findings of this study are available upon request from the corresponding author.

##### Conflict of interest

All authors declare no conflict of interest.

##### Funding

This research did not receive any specific grant from funding agencies in the public, commercial, or not-for-profit sectors.

## References

## References

[ref1] Abdel-Majid RM, Marshall JS (2004). Prostaglandin E2 induces degranulation-independent production of vascular endothelial growth factor by human mast cells. J Immunol.

[ref2] Agrawal AA (2015). Gingival enlargements: differential diagnosis and review of literature. World J Clin Cases.

[ref3] Baddireddy SM, Akula ST, Nagilla J, Manyam R (2022). Quantification of mast cells in oral reactive lesions: an immunohistochemical study. Acta Biomed.

[ref4] Cannavò SP, Bertino L, Di Salvo E, Papaianni V, Ventura-Spagnolo E, Gangemi S (2019). Possible roles of IL-33 in the innate-adaptive immune crosstalk of psoriasis pathogenesis. Mediators Inflamm.

[ref6] Gao Y, Min Q, Li X, Liu L, Lv Y, Xu W (2022). Immune system acts on orthodontic tooth movement: cellular and molecular mechanisms. Biomed Res Int.

[ref7] Gawish A, Gamal-Eldeen AM, Sherif SH, Neamat A (2010). Influence of etiological factors for gingival enlargement on some angiogenic and inflammatory mediators: an immunohistochemical study. J Am Sci.

[ref8] Gursoy UK, Sokucu O, Uitto VJ, Aydin A, Demirer S, Toker H (2007). The role of nickel accumulation and epithelial cell proliferation in orthodontic treatment-induced gingival overgrowth. Eur J Orthod.

[ref9] Hostynek JJ (2006). Sensitization to nickel: etiology, epidemiology, immune reactions, prevention, and therapy. Rev Environ Health.

[ref10] Huang S, Lu F, Chen Y, Huang B, Liu M (2013). Mast cell degranulation in human periodontitis. J Periodontol.

[ref11] Jain A, Jaiswal S, Vikey A, Bagulkar B, Bhat A, Shujalpurkar A (2021). Quantification of mast cells in nonreactive and reactive lesions of gingiva. J Oral Maxillofac Pathol.

[ref12] Jurela A, Verzak Ž, Brailo V, Škrinjar I, Sudarević K, Janković B (2018). Salivary electrolytes in patients with metallic and ceramic orthodontic brackets. Acta Stomatol Croat.

[ref14] Kandere-Grzybowska K, Letourneau R, Kempuraj D, Donelan J, Poplawski S, Boucher W (2003). IL-1 induces vesicular secretion of IL-6 without degranulation from human mast cells. J Immunol.

[ref15] Khatri MJ, Desai RS, Mamatha GS, Kulkarni M, Khatri J (2013). Immunohistochemical expression of mast cells using c-Kit in various grades of oral submucous fibrosis. ISRN Dermatol.

[ref16] Koch P, Bahmer FA (1999). Oral lesions and symptoms related to metals used in dental restorations: a clinical, allergological, and histologic study. J Am Acad Dermatol.

[ref17] Krystel-Whittemore M, Dileepan KN, Wood JG (2015). Mast cell: a multifunctional master cell. Front Immunol.

[ref18] Löe H, Silness J (1963). Periodontal disease in pregnancy I: prevalence and severity. Acta Odontol Scand.

[ref19] Lin TJ, Issekutz TB, Marshall JS (2000). Human mast cells transmigrate through endothelial monolayers and produce IL-8. J Immunol.

[ref20] Marjanović D, Andjelković Z, Brkić Z, Videnović G, Šehalić M, Matvjenko V (2016). Quantification of mast cells in different stages of periodontal disease. Vojnosanit Pregl.

[ref21] Mazreah SA, Shahsavari M, Kalati PA, Mazreah HA (2020). Immunohistochemical evaluation of CD117 in mast cells of aggressive periodontitis. J Indian Soc Periodontol.

[ref22] Mikulewicz M, Chojnacka K (2010). Trace metal release from orthodontic appliances: a systematic review. Biol Trace Elem Res.

[ref23] Nakayama T, Mutsuga N, Yao L, Tosato G (2006). Prostaglandin E2 promotes release of MCP-1 from mast cells. J Leukoc Biol.

[ref24] Novak-Bilić G, Vučić M, Japundžić I, Meštrović-Štefekov J, Stanić-Duktaj S, Lugović-Mihić L (2018). Irritant and allergic contact dermatitis. Acta Clin Croat.

[ref25] Patel N, Mohammadi A, Rhatigan RA (2012). Comparative analysis of mast cell quantification in dermatoses. ISRN Dermatol.

[ref26] Sheethal HS, Kn H, Smitha T, Chauhan K (2018). Role of mast cells in oral inflammatory and reactive pathologies. J Oral Maxillofac Pathol.

[ref27] Shimada Y, Hasegawa M, Kaburagi Y, Hamaguchi Y, Komura K, Saito E (2003). L-selectin or ICAM-1 deficiency reduces hypersensitivity response. J Immunol.

[ref28] Smith NR, Womack CA (2014). Matrix approach to guide IHC-based biomarker development. J Pathol.

[ref29] Spoorthi BR, Vidya GS (2013). Mast cell count analysis in oral lesions and malignancies. Int J Sci Res Publ.

[ref30] Tamir H, Theoharides TC, Gershon MD, Askenase PW (1982). Serotonin storage in mast cells. J Cell Biol.

[ref31] Theoharides TC, Bondy PK, Tsakalos ND, Askenase PW (1982). Differential release of serotonin and histamine. Nature.

[ref32] Theoharides TC, Kempuraj D, Tagen M, Conti P, Kalogeromitros D (2007). Mast cell mediators in inflammation. Immunol Rev.

[ref33] Tkaczyk C, Horejsi V, Iwaki S, Draber P, Samelson LE, Satterthwaite AB (2004). NTAL phosphorylation in mast cell activation. Blood.

[ref34] Trombetta D, Mondello MR, Cimino F, Cristiani M, Pergolizzi S, Saija A (2005). Toxic effects of nickel on oral epithelium. Toxicol Lett.

[ref35] Welle M (1997). Development and heterogeneity of mast cells. J Leukoc Biol.

[ref36] Williams CM, Galli SJ (2000). Mast cells amplify airway inflammation. J Exp Med.

[ref37] Yamazaki S, Nakano N, Honjo A, Hara M, Maeda K, Nishiyama C (2015). Role of transcription factor Ehf in mast cells. J Immunol.

[ref38] Zhang Z, Ernst PB, Kiyono H, Kurashima Y (2022). Utilizing mast cells in inflammatory diseases. Front Immunol.

[ref39] Zigante M, Spalj S, Prpic J, Pavlic A, Katic V, Matusan Ilijas K (2023). Gingival enlargement and metal allergy during orthodontic treatment. Materials (Basel).

